# Induction of apoptosis of human primary osteoclasts treated with extracts from the medicinal plant *Emblica officinalis*

**DOI:** 10.1186/1472-6882-8-59

**Published:** 2008-10-30

**Authors:** Letizia Penolazzi, Ilaria Lampronti, Monica Borgatti, Mahmud Tareq Hassan Khan, Margherita Zennaro, Roberta Piva, Roberto Gambari

**Affiliations:** 1 BioPharmaNet, ER-GenTech, Department of Biochemistry and Molecular Biology, Ferrara University, Ferrara, Italy; 2 Department of Pharmacology, Institute of Medical Biology, University of Tromso, Tromso, Norway; 3 H.E.J. Research Institute of Chemistry, International Center for Chemical Sciences, University of Karachi, Karachi, Pakistan; 4 Biotechnology Center, Ferrara University, Ferrara, Italy

## Abstract

**Background:**

Osteoclasts (OCs) are involved in rheumatoid arthritis and in several pathologies associated with bone loss. Recent results support the concept that some medicinal plants and derived natural products are of great interest for developing therapeutic strategies against bone disorders, including rheumatoid arthritis and osteoporosis. In this study we determined whether extracts of *Emblica officinalis *fruits display activity of possible interest for the treatment of rheumatoid arthritis and osteoporosis by activating programmed cell death of human primary osteoclasts.

**Methods:**

The effects of extracts from *Emblica officinalis *on differentiation and survival of human primary OCs cultures obtained from peripheral blood were determined by tartrate-acid resistant acid phosphatase (TRAP)-positivity and colorimetric MTT assay. The effects of *Emblica officinalis *extracts on induction of OCs apoptosis were studied using TUNEL and immunocytochemical analysis of FAS receptor expression. Finally, *in vitro *effects of *Emblica officinalis *extracts on NF-kB transcription factor activity were determined by gel shift experiments.

**Results:**

Extracts of *Emblica officinalis *were able to induce programmed cell death of mature OCs, without altering, at the concentrations employed in our study, the process of osteoclastogenesis. *Emblica officinalis *increased the expression levels of Fas, a critical member of the apoptotic pathway. Gel shift experiments demonstrated that *Emblica officinalis *extracts act by interfering with NF-kB activity, a transcription factor involved in osteoclast biology. The data obtained demonstrate that *Emblica officinalis *extracts selectively compete with the binding of transcription factor NF-kB to its specific target DNA sequences. This effect might explain the observed effects of *Emblica officinalis *on the expression levels of interleukin-6, a NF-kB specific target gene.

**Conclusion:**

Induction of apoptosis of osteoclasts could be an important strategy both in interfering with rheumatoid arthritis complications of the bone skeleton leading to joint destruction, and preventing and reducing osteoporosis. Accordingly, we suggest the application of *Emblica officinalis *extracts as an alternative tool for therapy applied to bone diseases.

## Background

Osteoclasts (OCs) are multinucleated cells of hematopoietic origin and are the primary bone resorbing cells [1-4]. There is increasing evidence that OCs play a crucial role in bone loss in rheumatoid arthritis [5-9], as recently reported by Ochi et al. [5] and reviewed by several authors, including Schett [6], Haynes [7], Tremoulet and Albani [8], Boyce et al. [9], Sato and Takayanagi [10] and Teitelbaum [11]. Abundant osteoclasts are found within the synovial tissue at sites adjacent to bone, creating resorption pits and local bone destruction followed by degradation of the bone matrix and calcium solubilization [6]. The basis of this behavior is that the synovial tissue of inflamed joints harbor high concentrations of monocytes/macrophages, which are osteoclast precursors, as well as cells that provide the specific molecular signals that drive osteoclast formation [6]. For instance, human rheumatoid synovial lymphocytes and fibroblasts promote osteoclastogenic activity by activating the receptor activator of NF-kB ligand (RANKL). The cytokines involved in this process are well known and have been the object of several studies [12,13], pointing out that TNFα, and IL-7 are involved in OCs differentiation [13]. Osteoclasts thus represent a link between joint inflammation and structural damage [6]. Hence, therapeutic approaches inhibiting osteoclastogenesis have been proposed by several authors for rheumatoid arthritis therapy [14-20].

These drugs are also expected to be of interest in the therapy of other pathologies associated with bone loss, such as osteoporosis [21-23] and bone metastasis [24-27], as inhibition of bone resorption, aimed at preventing further bone loss, is based on the efficient targeting of OCs function [21-27]. In conclusion, several therapeutic approaches are based on inhibition of osteoclast-dependent bone resorption through inhibition of OCs differentiation or stimulation of OCs apoptosis.

Recent results support the concept that some medicinal plants and natural products derived from them are of great interest for developing therapeutic strategies against bone disorders, including rheumatoid arthritis and osteoporosis [28-33]. Yin J. et al. demonstrated that water extracts of *Dioscorea spongiosa *stimulate osteoblasts proliferation, exhibiting at the same time a potent inhibitory activity on osteoclastogenesis [34]. Fruit extracts of *Psoralea corylifolia *[35] and *Cnidium monnieri *[36] have been shown to exhibit osteoblastic proliferation stimulating activity in osteoblast-like UMR106 cells *in vitro*. Several plant extracts inhibiting OCs differentiation also display strong anti-inflammatory properties [34-36].

*Emblica officinalis *is certainly a medicinal plant of interest [37-45]. It has played an important medicinal role for centuries in the Indian system of medicine. Fruits of *E. officinalis *are used for the treatment of a number of diseases, such as dyslipidemia [37] and atherosclerosis [38], as hepatoprotective [39], antibacterial [40] and anti-inflammatory agent [41]. In many cases, *E. officinalis *has been shown to be a potent free radical scavenging agent thereby preventing carcinogenesis and mutagenesis [42].

In this study we investigated whether extracts of *Emblica officinalis *fruits display effects of possible interest for the treatment of rheumatoid arthritis and osteoporosis, by activating programmed cell death of human primary osteoclasts. The effects of *E. officinalis *on human osteoclasts obtained from peripheral blood mononuclear cells [46,47] were analyzed by determining the proportion of apoptotic OCs. The effects of *Emblica officinalis *extracts were studied also on NF-kB/DNA interactions by electrophoretic mobility shift assay [48], given the possible involvement of the NF-kB transcription factor on the maintenance of the differentiation program of osteoclasts [49-51]. The effects of *Emblica officinalis *extracts on the expression of NF-kB dependent genes were also determined.

## Methods

### Culture of human primary osteoclasts

Human OCs were prepared as reported by Mitsuzaki et al. [52] with slight modifications. Peripheral blood was collected from healthy normal volunteers after informed consent. Mononuclear cells (PBMCs) were prepared from diluted peripheral blood (1:2 in Hanks Balanced Salt Solution), which was layered over Histopaque 1077 (Sigma, St. Louis, MO, USA) solution, centrifuged (400 g), washed and suspended in D-MEM/10% FCS. 3 × 10^6 ^PBMCs/cm^2 ^were plated in 24-well plates or in chamber slides and allowed to settled for 2 hours. Wells were then rinsed to remove non-adherent cells. Monocytes were maintained at 37°C, in 5% CO_2_, in medium supplemented with 10% FCS and cultured for 14 days in the presence of human M-CSF (25 ng/ml), RANKL (30 ng/ml) and 10^-7 ^M PHT. Culture media were replenished with fresh media every 3–4 days. Cells were used for the described experiments when mature multinuclear cells were predominant in the cultures.

### Tartrate-resistant acid phosphatase (TRAP) staining

TRAP staining of the cells was performed as reported by Villanova et al. [53]. Cells were fixed in 3% para-formaldehyde with 0.1 M cacodilic buffer, pH 7.2 (0.1 M Sodium cacodilate, 0.0025% CaCl_2_) for 15 min, extensively washed in the same buffer, and stained for TRAP (Acid Phosphatase Kit n. 386 – Sigma, St. Louis, MO, USA). After washing with distilled water and drying, mature TRAP positive multinucleated cells containing more than three nuclei were considered as osteoclasts.

### Plant extracts

The dried fruits of *Emblica officinalis *were extracted with absolute ethanol and the yield was 9.33%. The chemical composition has been determined by GC/MS and was reported elsewhere [54,55].

### Electrophoretic mobility shift assay (EMSA)

Electrophoretic mobility shift assay (EMSA) was performed by using double stranded ^32^P-labelled oligonucleotides as target DNA [51]. Binding reactions were set up as described elsewhere in binding buffer (10% glycerol, 0.05% NP-40, 10 mM Tris-HCl pH 7.5, 50 mM NaCl, 0.5 mM DTT, 10 mM MgCl2), in the presence of poly(dI:dC).poly(dI:dC) (Pharmacia, Uppsala, Sweden), 2 μg of crude nuclear extracts and 0.25 ng of labelled oligonucleotide, in a total volume of 20 μl [28]. After 30 min binding at room temperature, samples were electrophoresed at constant voltage (200 V for 1 hr) through a low ionic strength (0.25 × TBE buffer) (1 × TBE = 0.089 M Tris-borate, 0.002 M EDTA) on 6% polyacrylamide gels until the tracking dye (bromophenol blue) reached the end of a 16 cm slab. Gels were dried and exposed for autoradiography with intensifying screens at -80°C. In these experiments, DNA/protein complexes migrate through the gel with slower efficiency. In studies on the inhibitors of protein/DNA interactions, the addition of the reagents was as follows: (a) poly(dI:dC).poly(dI:dC); (b) labelled oligonucleotides mimicking the binding sites for transcription factors to be analyzed; (c) plant extracts; (d) binding buffer; (e) nuclear factors. The nucleotide sequences of double-stranded target DNA utilized in these experiments were 5'-CGC TGG GGA CTT TCC ACG G-3' (sense strand, HIV-NF-kB binding site), and 5'-CTG ATT TCC CCG AAA TGA CGG-3' (sense strand, STAT-3 binding site).

### Measurement of apoptosis

After 14 days of cell culture and 2–3 days of incubations with *E. officinalis *extracts, the cells were rinsed twice with PBS solution and fixed for 25 min in 4% paraformaldehyde at room temperature. Apoptotic cells were detected by the DeadEnd Colorimetric Apoptosis Detection System (Promega) according to the manufacturer's instructions. Measurement of apoptosis was calculated as a percentage of apoptotic nuclei (dark brown nuclei) versus total nuclei of multinucleated TRAP positive cells, evaluated in three independent measurements. A dark brown DAB signal indicates positive staining, while shades of blue-green to greenish tan indicate a nonreactive cell [46,47].

### Immunocytochemistry analysis

Immunocytochemistry analysis was performed employing the streptavidin-biotin method using Ultraystain Polyvalent-HRP Immunostaining Kit. OCs grown in multichamber slides were fixed in 100% cold methanol, and permeabilized with (v/v) Triton X-100 (Sigma) in TBS (Tris-buffered saline). Cells were incubated in 3% H_2_O_2 _and blocked with Super Block reagent (Ultraystain Polyvalent-HRP Immunostaining Kit). After the reaction with the primary antibodies, rabbit polyclonal antibodies of human origin (Santa Cruz Biotech) against MMP9, FAS receptor, IL-6, and NF-kB (2 mg/ml) were used accordingly to the manufacturer's protocols, at 1:500 (MMP9), 1:100 (FAS receptor), 1:800 (IL-6) and 1:800 (NF-kB) dilutions. Incubation was carried out at 4°C for 16 hr. Cells were then incubated at room temperature with anti-polyvalent Biotinylated Antibody (Ultraystain Polyvalent-HRP Immunostaining Kit). After rinsing in TBS, Streptavidin HRP (Ultraystain Polyvalent-HRP Immunostaining Kit) was applied, followed by the addition of Substrate-chromogen mix (AEC Cromogeno kit). After washing, cells were mounted in glycerol/TBS 9:1 and observed using a Leitz microscope [46,47].

### Cytotoxicity studies

The cytoxicity analysis was determined on *in vitro *cultured human OCs. PBMCs were plated in 96-well plates and, after 14 days, OCs were incubated with *E. officinalis *plant extracts for 3 days. Determinations of viable cells were performed after colorimetric assay with MTT (thiazolyl blue). The assay, based on the conversion of the yellow tetrazolium salt MTT to purple formazan crystals by metabolically active cells [56], provides a quantitative determination of viable cells. After 72 hr of treatments in triplicate, 200 μL of MTT was added to each well of cells, and the plate was incubated for 2 hr at 37°. The medium was removed, and the MTT crystals were solubilized with 50% DMF. Spectrophotometric absorbance of each sample was then measured at 570 nm.

### Statistical analysis

Data are presented as the mean ± SEM from at least three independent experiments. Statistical analysis was performed by one-way analysis of variance followed by the Student's t-test. A P value < 0.005 was considered statistically significant.

## Results

### Effects of *Emblica officinalis *extracts on differentiation and viability of human primary osteoclasts

**Figure 1:**
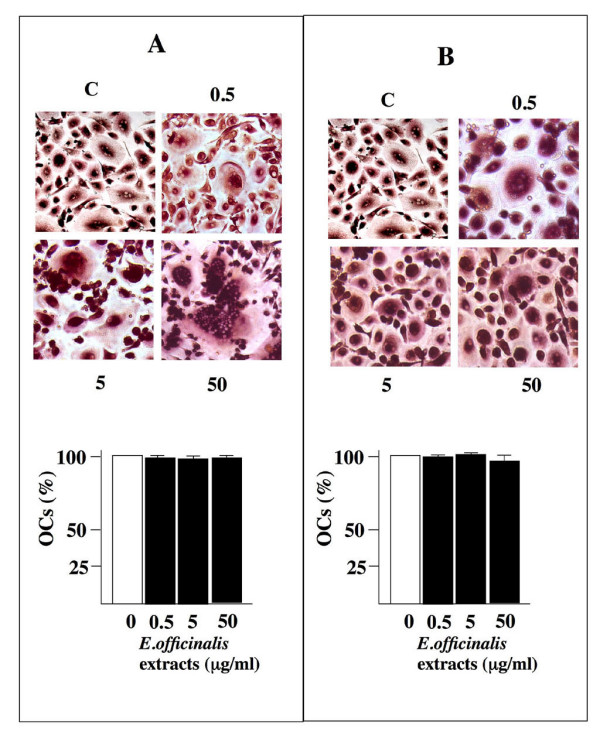
**TRAP staining analysis of human primary osteoclasts obtained after 14 days of culture in presence of 0.5, 5, and 50 μg/ml of *Emblica officinalis *extracts, as indicated (A); the same percentage of multinucleated TRAP-positive cells was obtained when mature osteoclasts were grown for 60 hours with the same amount of *Emblica officinalis *extracts (B).** Cells were photographed at the 20 × magnification. In the lower part of the panel data from five determinations are presented (average ± SD).

**Figure 2:**
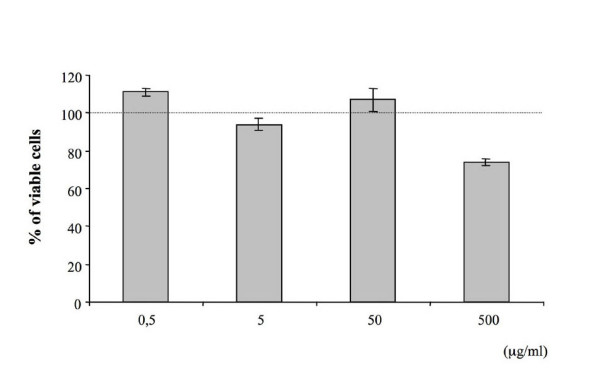
**Effect of different concentrations (0.5–500 μg/ml) of *Emblica officinalis *extract on cell survival of human primary osteoclast obtained by MTT colorimetric assay.** Results are expressed as the percentage of surviving cells and are the average ± SD of three independent experiments. The viability of controls only treated with vehicle (H_2_O) has been set as 100%.

Human primary osteoclasts were obtained from peripheral blood and cultured in complete D-MEM plus MCSF, PTH and RANKL for 14 days. OCs differentiation was tested by tartrate-acid resistant acid phosphatase (TRAP)-positivity (Fig. 1) and metalloproteinase-9 (MMP-9) expression (data not shown). In order to test the effect of *E. officinalis *extracts on osteoclast differentiation, mature OCs (Fig. 1A) or monocytes during the two weeks of induction (Fig. 1B) were exposed to 0.5, 5, 50 μg/ml of plant extracts. The conditions used for these experiments correspond to the concentrations of *E. officinalis *extracts leading to 50% of inhibition (IC_50 _value) of cell growth, previously analyzed in different cell lines [55]. As reported in Figure 1, the presence of comparable levels of TRAP-positive cells cultured both in presence and in absence of *E. officinalis *extracts did not affect the process of osteoclastogenesis, at the concentrations employed. Quantitative data from three independent experiments are presented in the lower sides of Figure 1, demonstrating that treatment of the cultures with *E. officinalis *extracts does not have inhibitory effects on the development of TRAP-positive OCs. Cytotoxic effects of *E. officinalis *extracts were then analyzed. Human primary OCs were treated with increasing amount of *E. officinalis *extracts (0.5–500 μg/ml) for 72 hours and the viability of the cells was examined by the colorimetric MTT assay [56]. As shown in Figure 2, 0.5, 5 and 50 μg/ml of *E. officinalis *extracts did not cause any cytotoxic effect on the total cell population (1–5% of which is constituted by OCs). Only *E. officinalis *extracts used at 500 μg/ml were found to induce a slight but not significant decrease of viability.

### *Emblica officinalis *extracts induce apoptosis of osteoclasts

**Figure 3:**
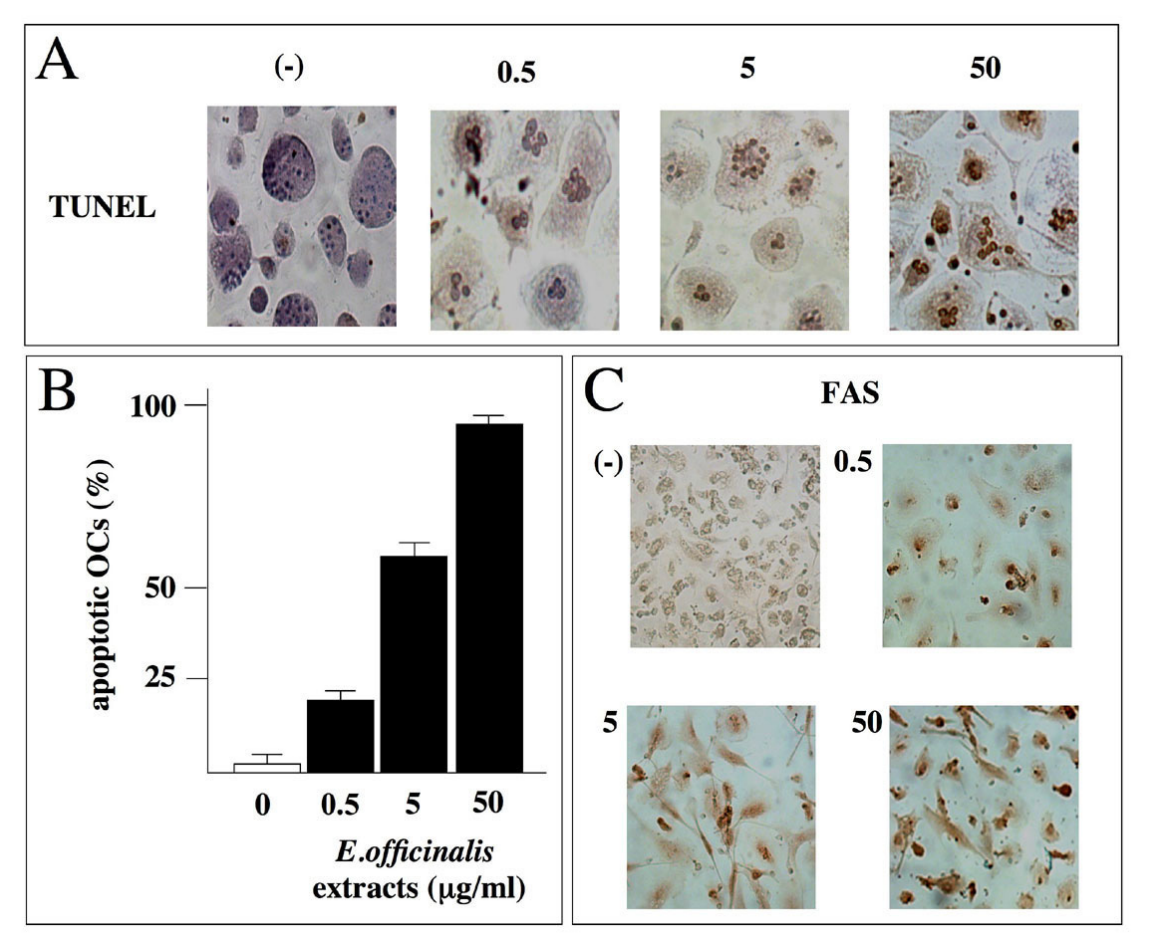
**A, B. Detection of apoptosis by TUNEL assay in human primary osteoclasts. **The presence of apoptotic OCs after treatment with 0.5, 5, 50 μg/ml of *Emblica officinalis *extract for 60 hours is shown in panel A. Brown color reaction indicates cells that underwent apoptosis. Quantitative results of the experiment shown in panel A are depicted in panel B (the data reported represent the average ± SD of six independent determinations). C. Immunocytochemical analysis of FAS receptor expression levels in human primary osteoclasts subjected to the same experimental conditions reported in panel A. (-): control cells. Cells were photographed at the 20 × magnification.

In a previous study we demonstrated that in different cell lines (K562, B-lymphoid Raji, T-lymphoid Jurkat and HEL cells) *E. officinalis *extracts retain an antiproliferative effect [55]. In the present paper we investigated osteoclasts in terms of apoptosis. To this aim, TUNEL test was performed on OCs after exposure, up to 60 hours, to 0.5, 5, and 50 μg/ml of *E. officinalis *extracts. As shown in the representative experiment reported in Figure 3 (panels A and B), a low but significant level of apoptosis (20%) was induced by 0.5 μg/ml of extract; at 5 and 50 μg/ml, a dramatic increase (respectively 50% and 98%) in TUNEL-positive nuclei was observed. Table 1 reports summary data from three independent experiments, confirming the observation that 5 and 50 μg/ml of *E. officinalis *extracts consistently induce high levels of apoptosis of osteoclasts. Times of exposure shorter than 60 hours were also tested (24 and 48 hours) without obtaining significant differences from untreated cells (data not shown). These results were confirmed by immunocytochemical analysis of FAS receptor, a well known apoptosis-related protein [57], whose expression increased, as shown in Figure 3C, in OCs treated with *E. officinalis *extracts at all the concentrations used. When extracts from different medicinal plants, such as *Satureja montana *and *Satureia hortensis *were employed, no OCs apoptosis was induced (data not shown).

**Table 1:** Apoptotic osteoclasts following treatment with *E. officinalis *extracts

Experiment	*E. officinalis *extracts (μg/ml)			
	0	0.5	5	50
A	2	20	33	97
B	3	15	58	98
C	2.5	55	65	99

Detection of apoptosis of human primary OCs was performed by TUNEL assay. Treatment with 0.5, 5, 50 μg/ml of *Emblica officinalis *extracts was carried on for 60 hours. See Figure 3 for additional information.

### *In vitro *effects of *Emblica officinalis *extracts on NF-kB transcription factor activity

**Figure 4:**
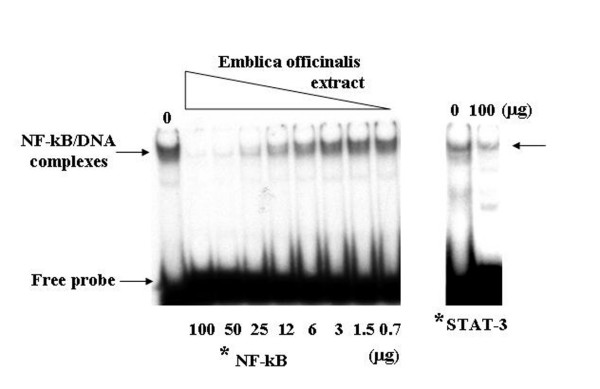
**Analysis by electrophoretic mobility shift assay of the effects of *Emblica officinalis *extracts on NF-kB DNA binding activity.** Nuclear extracts from the K562 cell line were incubated with ^32^P-labelled oligonucleotides (*) NF-kB and STAT-3, in the presence of different amounts (100, 50, 25, 12.5, 6, 3, 1.5, 1 μg) of *Emblica officinalis *extracts. Protein/DNA complexes and free probe are indicated by arrows.

The ability of *E. officinalis *extracts to interfere with NF-kB binding to DNA was investigated, given that the transcription factor NF-kB plays a critical role in OCs activities by regulating the expression of a large number of OCs specific genes [48-50]. *E. officinalis *extracts were incubated in presence of 5 μg of nuclear extracts from K562 cells with an oligonucleotide containing a cis element of the LTR of HIV-1 representing the DNA binding site for NF-kB. DNA-protein interactions were then analyzed by EMSA [51]. As reported in Figure 4, a dose dependent effect was observed, indicating the ability of *E. officinalis *extracts to completely inhibit NF-kB interaction with its cis element, when used at 100, 50, and 25 μg/reaction. On the contrary, 100 μg of *E. officinalis *extracts were not able to abolish the DNA-protein interactions of the transcription factor STAT-3 with its cis element (right side of Fig. 4) indicating a selectivity of the effects of *E. officinalis *extracts for NF-kB/DNA interactions. The sensitivity of NF-kB/DNA interactions to *E. officinalis *extracts was demonstrated to be related to the type of plant extracts and not to the extracting buffers, since no inhibitory effects were observed (a) with the extracting buffer and (b) other extracts from medicinal plants, such as *Oroxylum indicum, Cuscuta reflexa, Paederia foetida, Hygrophilla auriculata, Ocimum sanctum *(data not shown and Lampronti et al.) [57].

**Figure 5:**
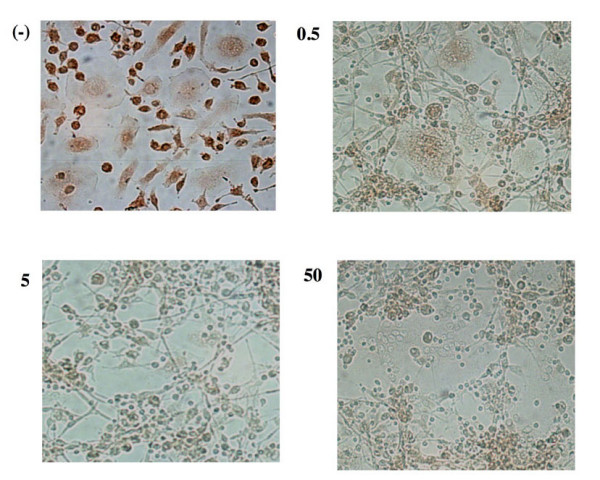
**Different expression of IL-6 in human primary osteoclasts analyzed by immunocytochemistry with specific antibody after incubation for 60 hours in the absence (-), or in the presence of 0.5, 5 and 50 μg/ml of *Emblica officinalis *extracts.** Cells were photographed at the 20 × magnification.

In order to determine whether *E. officinalis *extracts affect NF-kB dependent biological activity in OCs, we have evaluated the effects of the plant extracts on the expression of IL-6, a target gene of NF-kB transcription factors [58,59]. Immunocytochemical analysis, reported in Figure 5, clearly shows a significant decrease of IL-6 levels in OCs treated with 5–50 μg/ml of *E. officinalis *extracts after comparison to control untreated cells.

## Discussion

The present study suggests the employment of primary cultures of human osteoclasts as a tool to test the potential interest of extracts of *Emblica officinalis *fruits in the experimental therapy of human pathologies associated with bone loss, including osteoarthritis and osteoporosis. The possible use of natural products, including plant extracts and nutriaceuticals, is under debate. A systematic review of the scientific evidence supporting the hypothesis that nutrition can improve the symptoms of declared osteoarthritis has been recently published [60]. In addition, the possible use of medicinal plant extracts or single products derived from them for preventing or treating experimentally rheumatoid arthritis has been reported [31-33]. In this respect, one of the best example is Turmeric, derived from the plant *Curcuma longa*, a gold-colored spice commonly used in the Indian subcontinent in Ayurvedic medicine as a treatment for inflammatory disorders, including arthritis. On the basis of this traditional usage, dietary supplements containing turmeric rhizome and curcuminoid-containing turmeric extracts are used in the western world for arthritis treatment and prevention [31].

*Emblica officinalis *is reported to have antitumor activity [42,45,54] together with beneficial effects in gynecological, hepatic, respiratory and skin [43,44] disorders. However, the biological activity of *Emblica officinalis *extracts of possible interest for treatment of arthritis and osteoporosis have not yet been reported. To verify this effect *in vitro *different experimental approaches should be used in parallel, including analysis of possible positive effects on osteoblastogenesis and negative effects on osteoclastogenesis. In fact, increase of bone formation, leading to anti-osteoporotic and anti-osteoarthritis activity, could be obtained by induction of osteoblast activity, inhibition of osteoclast bone resorption, or both of these effects. In previous studies [34-36] osteoblast-like UMR106 cells, derived from a rat osteogenic sarcoma, were used to screen drugs and plant extracts for stimulation of bone formation.

The aim of our study was to determine the activity of *E. officinalis *extracts on osteoclasts, using primary OCs of human origin isolated from peripheral blood and incubated for different length of time and with different amounts of *E. officinalis *extracts. The analysis of cellular viability and apoptosis demonstrates that these plant extracts do not have any cytotoxic effect, even at a concentration of 500 μg/ml, still inducing significant level of apoptosis. This effect was confirmed by the finding of increasing levels of FAS receptor after treatment with both high and low concentrations of *E. officinalis *extracts. Hence, we conclude that *E. officinalis *extracts are strong inducers of the apoptotic pathway of primary human osteoclasts.

Since the transcription factor NF-kB has been reported to be important for the expression of several osteoclast-specific genes, we verified whether *E. officinalis *extracts were able to inhibit the biological activity of this factor. An electrophoretic mobility shift assay demonstrated that NF-kB/DNA complexes are inhibited after incubation of nuclear DNA-binding proteins with increasing amounts of *E. officinalis *extracts. Accordingly, when the analysis was carried on cultured OCs, high levels of inhibition of IL-6, a NF-kB modulated protein were found, further demonstrating that NF-kB dependent biological functions are impaired following treatment with *E. officinalis *extracts. On the basis of this experimental evidence we propose that the pro-apoptotic action of *E. officinalis *extracts on osteoclasts could be mediated, at least in part, by interfering with NF-kB activity.

Interestingly, the effects on human OCs of *E. officinalis *extracts are similar to those reported for other inhibitors of NF-kB functions, such as biphenylcarboxylic acid butanediol ester (ABD56) [61] and genistein [62]. In addition the effects of *E. officinalis *extracts are almost over imposable to those of a decoy double-stranded oligonucleotide mimicking NF-kB binding sites [46]. Similarly to this decoy oligonucleotide, *E. officinalis *extracts, at the concentrations employed, induce OCs apoptosis without inhibiting osteoclastogenesis. An effect of *E. officinalis *at higher concentrations cannot be excluded; however, at these levels a certain cytotoxicity (see Figure 2) renders difficult the discrimination between a possible inhibitory effect on OCs differentiation and an overall antiproliferative activity.

Finally, we like to point out that our results are based on an *in vitro *approach, and specificity *in vivo *of the effects here described should be carefully determined, since the transcription factor NF-kB is also important for other cellular systems, including macrophages, that when exposed to the extracts may overwhelm the effects on osteoclasts. In this respect, several in vivo systems suitable for testing inducers of OCs apoptosis are now available [63-65].

## Conclusion

The data here reported on the effects of *E. officinalis *extracts on mature human osteoclasts suggest the possible use of this medicinal plant as a therapeutic tools against different forms of arthritis and osteoporosis, improving the activity of already employed drugs. In addition, *E. officinalis *extracts could be analyzed to identify single compounds responsible for the biological activity identified.

## Abbreviations

OCs: osteoclasts; TRAP: tartrate-resistant acid phosphatase; NF-kB: nuclear factor kappa-B; RANKL: receptor activator of NF-kB ligand; PBMCs: peripheral blood mononuclear cells; PTH: parathyroid hormone; MCSF: macrophage colony-stimulating factor; MTT: 3-(4,5-Dimethylthiazol-2-yl)-2,5-diphenyltetrazolium bromide.

## Competing interests

The authors declare that they have no competing interests.

## Authors' contributions

LP developed the culture of osteoclasts from the peripheral blood; IL characterized the *E. officinalis *extracts with respect to effects of cell growth; MB performed gel shift experiments using nuclear factors and oligonucleotides mimicking the NF-kB binding sites; MTHK isolated the the *E. officinalis *extracts; RP designed the experiments with the osteoclasts cultures; RG wrote the paper and coordinated the experiments. All authors read and approved the final manuscript.

## Pre-publication history

The pre-publication history for this paper can be accessed here:


